# The Common Cold and Influenza in Children: To Treat or Not to Treat?

**DOI:** 10.3390/microorganisms11040858

**Published:** 2023-03-28

**Authors:** Natalia A. Geppe, Andrey L. Zaplatnikov, Elena G. Kondyurina, Maria M. Chepurnaya, Natalia G. Kolosova

**Affiliations:** 1 Filatov Clinical Institute of Children’s Health, Sechenov First Moscow State Medical University, Moscow 119435, Russia; 2 Russian Medical Academy of Continuous Professional Education, Moscow 123242, Russia; 3 Department of Pediatrics, Faculty of Advanced Training and Professional Retraining, Novosibirsk State Medical University, Novosibirsk 630091, Russia; 4 Pulmonology Department, Rostov Regional Children’s Clinical Hospital, Rostov 344015, Russia; 5 Department of Pediatrics, Rostov State Medical University, Rostov 344022, Russia

**Keywords:** common cold, acute respiratory viral infection, treatment, antibody-based drugs

## Abstract

The common cold, which is mostly caused by respiratory viruses and clinically represented by the symptoms of acute respiratory viral infections (ARVI) with mainly upper respiratory tract involvement, is an important problem in pediatric practice. Due to the high prevalence, socio-economic burden, and lack of effective prevention measures (except for influenza and, partially, RSV infection), ARVI require strong medical attention. The purpose of this descriptive literature review was to analyze the current practical approaches to the treatment of ARVI to facilitate the choice of therapy in routine practice. This descriptive overview includes information on the causative agents of ARVI. Special attention is paid to the role of interferon gamma as a cytokine with antiviral and immunomodulatory effects on the pathogenesis of ARVI. Modern approaches to the treatment of ARVI, including antiviral, pathogenesis-directed and symptomatic therapy are presented. The emphasis is on the use of antibody-based drugs in the immunoprophylaxis and immunotherapy of ARVI. The data presented in this review allow us to conclude that a modern, balanced and evidence-based approach to the choice of ARVI treatment in children should be used in clinical practice. The published results of clinical trials and systematic reviews with meta-analyses of ARVI in children allow us to conclude that it is possible and expedient to use broad-spectrum antiviral drugs in complex therapy. This approach can provide an adequate response of the child’s immune system to the virus without limiting the clinical possibilities of using only symptomatic therapy.

## 1. Common Cold or Acute Respiratory Viral Infections: Causative Agents

The COVID-19 pandemic has highlighted the problem of treatment of acute respiratory viral infections (ARVI), often referred to as the “common cold” [1,2]. These infections are caused by so-called “seasonal” pathogens—RNA (orthomyxoviruses, paramyxoviruses, coronaviruses, etc.) and DNA (adenoviruses, bocaviruses, etc.) viruses that target the respiratory tract. The “seasonality” of ARVI pathogens depends on the climate zone [3,4]. On this basis, all ARVI pathogens are divided into three groups [5]. The incidence of “winter” viruses reaches its peak in mild-climate countries in winter and includes influenza A virus (IАV), influenza B virus (IBV), coronaviruses (CoV) (strains OC43, HKU1, 229E, and NL63), and respiratory-syncytial virus (RSV). Activity of certain enteroviruses (EV, but not rhinoviruses (RV)) increases during the summer; thus, they are referred to as “summer” viruses. Adenovirus (ADV), human bocavirus (BoV), human parainfluenza virus (PIV), human metapneumovirus (MPV), and RV are considered “year-round” viruses. The seasonality of PIV depends on its type—hPIV type 1 peaks in autumn, while hPIV type 3 peaks in spring and summer.

In children, the most frequently observed ARVI pathogens are RV, IАV/IBV, PIV, RSV, CoV, MPV, and BoV [6,7,8,9,10]. The spectrum of ARVI pathogens in children varies not only according to the time of year, but also according to age. A Chinese study published in 2021 retrospectively analyzed the results of oropharyngeal swabs in 103,210 children with ARVI in the outpatient department of the Children’s Hospital of Zhejiang University School of Medicine. The results of this study showed that the detection rate of ADV was highest in preschool children, RSV was highest in infants, and the incidence of influenza increased with age [11].

The COVID-19 pandemic impacted the epidemiology of ARVI. Although non-pharmacological prophylaxis has not completely stopped their spread, it should be noted that in certain countries these measures have resulted in a significant yet temporary decrease in the frequency of respiratory infections in children [12,13,14,15,16,17]. In Poland, for example, there was a 100% and 99% decrease in the incidence of RSV and RV respiratory infections until May 2021, respectively. In August 2021 the incidence of RSV infections increased [12]. During the COVID-19 pandemic, a decrease in seasonal influenza virus (IV) activity in children was reported in the USA, England, Australia, Canada, South Africa, Chile, Singapore, Japan, and in a number of other countries [13,14,15,16,17].

## 2. The Problem of Asymptomatic ARVI

The course of ARVI in children varies from asymptomatic and mild respiratory signs such as rhinorrhea, nasal congestion, sneezing and other nasopharyngeal/upper laryngeal symptoms to severe bronchitis, bronchiolitis, and pneumonia [18].

The problem of asymptomatic ARVI deserves special attention. The problem of asymptomatic courses of ARVI deserve special attention, in particular, due to the fact that a sick child is a source of infection for other people, including family members. For example, the frequency of asymptomatic influenza, according to a study involving 1272 children aged 0 to 14 years without immunodeficiency, averaged 6.6%, increasing with age—1.7% under the age of 1 year, 3.5% in children from 2 to 4 years old and 9.1% in children from 5 to 14 years old (*p* < 0.001) [19].

In addition to an increase in the burden of ARVI, this leads to the infection being underdiagnosed. This has been clearly demonstrated in recent years by the example of COVID-19, where the number of asymptomatic cases could reach 55.6% among confirmed cases of SARS-CoV-2 infection [20].

## 3. The Role of the ARVI Pathogen in the Clinical Picture

Risk factors for severe respiratory infections in children include age (depending on the causative agent), prematurity, presence of chronic diseases, and, for some viruses, high viral load (e.g., RSV).

According to an 11-year prospective study of 4312 children hospitalized with community-acquired respiratory tract infections, the proportion of patients with a severe course of seasonal CoV was similar to that of RV (136 out of 987; 14%), and lower than that of RSV (338 out of 870; 39%) [8]. Another 14-year prospective study showed no significant clinical difference in the severity of ARVI in 5131 children hospitalized with seasonal CoV, RV, RSV, and influenza [21].

In addition, viral–viral and viral–bacterial associations, as well as combined respiratory and gastrointestinal tract involvement are features of the current course of ARVI in children [8,22,23,24,25]. The frequency of viral infections occurring simultaneously with bacterial pneumonia is currently 30–50% in both adults and children [26]. The presence of co-infections leads to difficulties in the diagnosis. It has been noted that signs of myocarditis, vasculitis, and electrocardiogram changes are more frequently observed in children after co-infection [24]. However, despite the adverse effects (AEs) of co-infection shown in studies, there is evidence of relatively positive viral–viral interactions. For example, the CoV and RV co-infection had low rates of severe ARVI courses, suggesting a modifying effect of RV on CoV [8]. Some data suggests that RV triggers interferon (IFN)-mediated activation of innate immune response, thus preventing even SARS-CoV-2 from entering cells [27].

The social burdens of ARVI in children include high rates of illness, which, although mild in most cases, result in missing school; absence from work due to the child’s illness; and, without proper attention, these can lead to serious health problems [28,29]. In a cohort study involving 6266 children, the proportion of children missing school/kindergarten due to ARVI varied from 21.4% (BoV) to 52.1% (IV) [30].

The high frequency of ARVI in children is related to the developmental features of the immune system [31]. Due to physiological deficiencies in the immune system, the course of ARVI can be severe and prolonged and can contribute to the development of chronic diseases, including allergic diseases.

It is believed that ARVI in children is a form of “training” for the immune system. However, due to its immaturity, complications occur during frequent and severe viral infections. Most complications affect the lower respiratory tract. Several studies were conducted to clarify the features of the epidemiology of ARVI during the COVID-19 pandemic. It was shown that a large proportion of children’s hospital admissions are still due to severe ARVI with RV and RSV being the leading cause. In a cohort study conducted in Australia and seven countries in Southeast Asia and Latin America involving 6266 children, the rate of ARVI-related hospitalizations ranged from 1.03% for BoV to 23.69% for RV/EV [30].

According to a study conducted in the USA involving 898 children under 5 years of age, RSV-positive children (*n* = 191, 28%) were more frequently admitted to an intensive care unit and more likely to require oxygen, compared to children with other viruses [32]. In a cohort study, pneumonia was registered as the cause of acute respiratory distress syndrome (ARDS) in more than half of the 544 children with ARDS [33]. Pneumonia had a viral etiology in 75.2% of cases, and the most common pathogens were RV (43% of cases), RSV (24%), MPV (17%), ADV (14%) and IV (13%). At the same time, viral co-infections were detected in a quarter of cases, 68% of which were caused by RSV [33].

The most common pathogens of acute bronchiolitis that lead to ARDS in children under 2 years old were RSV and RV (79%) [34]. Recently published results showed that seasonal CoV can also cause pneumonia in children [35]. In addition, a systematic review published in March 2022 with a meta-analysis of 38 studies showed that preschool children with RV–bronchiolitis were more likely to develop recurrent wheezing and bronchial asthma (BA) than preschool children with RSV–bronchiolitis, with an odds ratio (OR) of 4.11; a 95% confidential interval (CI) of 2.24–7.56 and an OR of 2.72; and a 95% CI of 1.48–4.99, respectively [36]. IV, RSV, RV, MPV, PIV, and BoV have also been shown to play a significant role in cases of wheezing and exacerbation of pre-existing BA in children [37,38].

## 4. Immune Response: Small but Important Accents

The key determining factor of the ARVI course in a child is the immune response to infection, which depends on both individual and environmental factors. Among them are the lack of immunological memory in young children, the lymphoepithelial pharyngeal ring being incompletely developed until 5–7 years of age, etc. [31]. The adaptive immune response is initially biased—starting as predominantly a Тreg and Th2 response, changing to a Th1 response by the end of the 1st–beginning of the 2nd year of age. The functionality and number of antigen-presenting cells at an early age are reduced, and intercellular cooperation and functional activity of phagocytes are disrupted [39]. In young children with ARVI, there is a 30% reduction in the functional activity of natural killer (NK) cells [40].

Age is a major factor that influences cytokine levels in a healthy pediatric population. Insufficiency of cytokine synthesis at an early age has been detected [39]. Therefore, in circumstances where the body requires an active innate immune protection mediated with interferons (IFNs) for an effective pathogen elimination, children have a weaker response compared to adults: the IFNs production by peripheral blood cells is reduced, in contrast to adults, by 9 times in children under the age of 1 year, and by 6 times in children from 1 to 3 years of age [31,40]. Interestingly, the antiviral immune response can potentially depend on the duration of breastfeeding. There is evidence that spontaneous production of IFNγ and interleukin (IL)-10 in whole blood culture is more pronounced in children who have been breastfed for more than 4 months [41].

The dysfunction of the local immune response in children is also worth noticing; the nasal secretion of children aged 6 months to 3 years old has significantly lower concentrations of secretory immunoglobulin A (sIgA) and lysozyme than that of adults. Full-fledged local immune response develops in children only by the age of 5–7 years [40].

Age-related differences in the immune response to infections are supported by studies illustrating the importance of age as a factor in the severity of ARVI in children. For example, a Norwegian study involving 171 children under 16 years old showed that the risk of severe ARVI is higher in children from 1 year of age for MPV infections and from 6 months of age for RSV infections, respectively [42].

Each causative agent in the pathogenesis implements its own direct and indirect mechanisms of cellular damage, but the immune response is characterized by universality. When encountering a respiratory virus, the innate immune response can be either adequate or incomplete, slowed or weakened, or delayed—in the form of an excessive response after a delay, which can lead to tissue damage [43]. This is particularly important in children with comorbidities and immunocompromised patients, including asthmatics [44,45]. In children with BA, the immune response is imbalanced and may lead to a mutually reinforcing effect of the allergic component of BA and the ARVI pathogen on the inflammation and exacerbation of respiratory tract lesions [45].

## 5. IFNɣ—Cytokine with Antiviral and Immunomodulatory Effects in ARVI Pathogenesis

The key antiviral protective factors, IFNs, deserve special attention as potential therapeutic targets in ARVI [46]. The antiviral and immunomodulatory effects provided by IFNs are crucial in ARVI. To cope with the virus, the body must not only limit the viral replication and initiate an antiviral immune response, but also regulate this response through negative feedback to minimize tissue damage. Because of their importance in pathogenesis, the role of IFNs in the resistance of viral aggression has been studied during the COVID-19 pandemic [47,48,49]. Type II IFN, represented by a single cytokine—IFNɣ, is unique in the system of antiviral defense [50]. IFNɣ plays an important role in both innate and adaptive immune response to the virus. Participating in the regulation of cell-mediated immune responses, IFNɣ is important for the function of type I IFNs—for example, macrophage activation and the enhancement of antigen presentation [51]. In turn, type I IFNs activate the NK cells and induce IFNɣ production. There are data on the dual nature of the mechanism of action of type I and II IFNs in the innate antiviral immune response [51,52]. A local synergistic effect of type I and II IFNs in the mucosa has also been demonstrated to limit viral replication [51]. Even suboptimal concentrations of IFNɣ, which do not activate immune cells, prepare them for a subsequent response to stimuli and rapid induction of an inflammatory response, as well as provide a refractory state to viral proliferation in the surrounding tissues as part of the important antiviral effector mechanisms [52]. Although IFNɣ may exert some pro-viral effect according to several studies, its multimodal role in antiviral protection mechanisms in ARVI as well as SARS-CoV-2 has been shown [52,53]. The ability to inhibit the attachment of IAV to the host cell by affecting sialic acid clustering as one of the antiviral mechanisms of IFNɣ was described.

A systematic review by authors from Switzerland, Germany, UK, Australia, and the Netherlands concluded that IFNɣ expression strongly depends on age, with its lowest levels in newborns and a subsequent increase with age. This aspect is associated with the stages of immune system maturation [54].

## 6. Approaches to the Treatment of ARVI

The main objectives of ARVI treatment in children include the virus’ elimination, a reduction of the severity and management of the symptoms, an adequate immune response to prevent complications, a chronicity of the infection, and the exacerbation of comorbidities. There is a very thin line between a protective immune response that limits viral replication and an imbalanced immune response with a dysregulated immune activity that leads to the most severe complications [55]. Therefore, the main target in ARVI treatment is to influence the “virus—immune response” functional system. This explains the constant search for drugs that can help to direct and modify the immune response as well as ensure its balance and adequacy, regardless of the etiological agent and concomitant pathology. The understanding of the molecular mechanisms of ARVI enables the development and use of more effective immune-mediated antiviral strategies in clinical practice.

Currently, there are various approaches to the treatment of ARVI—some countries recommend only symptomatic treatment, with the exception of influenza and RSV infection, while others in addition recommend a wide range of drugs with antiviral action, including those that can affect the immune system—so-called immunotropic drugs [56,57,58,59,60]. Immunotropic drugs focus primarily on supporting and providing antiviral immunity. They include immunomodulatory peptides, cytokines, antibody (Ab)-based drugs, including drugs based on affinity purified technologically processed antibodies (APTP Abs), synthetic immunomodulators, immunosuppressants, nucleic acids, drugs based on bacterial lysates, and herbal remedies [60].

The above-mentioned current statistics and epidemiological data make one wonder whether an approach to only the symptomatic treatment of ARVI without a timely and adequate antiviral therapy is justified or even dangerous.

## 7. Direct-Acting Antiviral Drugs for the Treatment of ARVI

A large number of ARVI causative agents and their ability to change, including drug resistance and high risks in the research and development of drugs, have led to the fact that there are only a few antiviral drugs for the treatment of ARVI. In routine practice, direct-acting antiviral drugs for the treatment of ARVI are presented only for infections caused by the IV. These include M2 ion-channel inhibitors (amantadine, rimantadine), neuraminidase inhibitors (oseltamivir, zanamivir, peramivir), CAP-dependent viral endonuclease inhibitors (baloxavir), nucleosides and their analogues (ribavirin, favipiravir), and several others [57,60,61,62,63]. Neuraminidase inhibitors are recommended for early treatment of influenza in children, as they reduce the duration of the illness, the severity of symptoms, and the risk of complications that lead to hospitalization and/or death [64]. However, current strains of IAV are known to have developed a resistance to the neuraminidase inhibitor oseltamivir, as well as an almost complete resistance to M2 ion channel inhibitors [65,66]. In addition, cases of serious AEs with oseltamivir have been reported in recent years. For example, one study described a sinus bradycardia in a 5-year-old patient taking oseltamivir for laboratory-confirmed influenza [67]. The other study described oseltamivir-related gastrointestinal bleeding and liver damage in a 6-year-old child [68].

The search for target molecules and potential drug substances for the treatment of ARVI is ongoing [28,49,69,70,71,72]. However, there are several limiting factors: variability in the pathogen species and the associated low efficacy due to the lack of broad-spectrum antiviral drugs, as well as many AEs. For example, for the leading causative agent of seasonal ARVI in children, RV, the vaccines are hampered by a large number of serotypes that produce only weakly cross-neutralizing Abs. In addition, the antiviral drugs that have been developed over many years have not received regulatory approval in different countries due to low efficacy and/or AEs [28]. In several countries, including the Russian Federation, drugs with an indirect action are widely used, and the term “immune-mediated broad-spectrum antiviral drugs” can be used to describe their mechanism of action. This group includes a fairly large pool of drugs with immunomodulatory properties [59].

## 8. Antibody-Based Drugs in the Immunoprophylaxis and Immunotherapy of ARVI

Modern technologies have led to the development of an innovative type of antivirals—Abs-based drugs [69,70]. The properties of monoclonal and polyclonal Abs, convalescent plasma, and other Ig-based drugs are studied in many countries to assess the possibility of their use as active pharmacological drug substances for the prevention and treatment of influenza and other ARVI [69,73,74,75]. The interest in Abs-based drugs is primarily due to the possibility of using the body’s natural defenses to minimize the potential harm/AEs of therapy.

The monoclonal Ab drug palivizumab and some candidate drugs (for example, motavizumab) can be used for the prevention and treatment of ARVI caused by RSV [69,76]. Palivizumab’s widespread use is limited by its high cost and the necessity that it be administrated monthly [77]. Therefore, the use of palivizumab is restricted to high-risk groups, including infants and young children [69]. The efficacy and safety of intravenous administration of polyclonal Ig, a human serum preparation containing high levels of a virus-neutralizing Abs, for the treatment of severe RSV infection has been studied [69]. Also in the 2019 Cochrane review, containing 7 randomized clinical trials (RCTs) that involved 486 children under 3 years old, polyclonal Ig and monoclonal Ab drugs palivizumab and motavizumab were compared to a placebo in the treatment of hospitalized patients with laboratory-confirmed lower respiratory tract RSV infections. The authors concluded there is insufficient evidence of differences in the mortality, duration of hospitalization, and safety between these Abs-based drugs and the placebo [78].

Drugs based on APTP Abs to IFNγ, including Anaferon for children and Ergoferon, are active against a wide spectrum of ARVI causative agents [59,79,80]. A systematic review with a meta-analysis of nine double-blind placebo-controlled RCTs that included data from 2790 patients older than 1 month showed that Anaferon for children, compared to placebo, significantly reduced the duration of the disease as well as the duration of individual symptoms in ARVI [79]. The weighted average effect size was 1.05 for the endpoint “duration of the disease” [95% CI 0.44; 1.67], and 0.97 for the endpoint “duration of fever” [95% CI 0.61; 1.33] (*p* < 0.001, *p*-value of the two-sided Z-test; model of random effects). The efficacy of Anaferon for children with ARVI did not depend on the causative agent and the presence of comorbidities. According to this meta-analysis, the preventive efficacy of Anaferon for children against ARVI pathogens, including patients with bronchopulmonary pathology and children who were often ill, was 1.3 times higher than that of a placebo (the criterion “the proportion of patients who did not get sick”: relative risk (RR) = 1.25 [95% CI 1.2; 1.3]; OR = 2.22 [95% CI 1.7; 2.9]) [79]. Data from a meta-analysis published in late 2021 demonstrated that Ergoferon is 1.5 times more effective in reducing the duration of fever compared to a placebo (ОR = 1.499, *р* = 0.0002). In addition, Ergoferon reduces the severity of the disease (area under the curve 32.83 ± 18.12 vs. 36.94 ± 19.08 conventional units in the placebo group, *p* = 0.0083) as well as the incidence of bacterial complications by seven times, compared to the placebo. The safety of the therapy was confirmed by the lower number of patients with AEs (0.6% vs. 10.8% in the placebo group, *p* = 0.0006) and the absence of drug–drug interactions [80].

Apart from that, the properties of human monoclonal Abs; which neutralized major circulating strains of IAV in vitro and in vivo, including the polyreactivity; are studied in the context of possible development of influenza vaccines and more effective antiviral drugs [75,81].

## 9. Antihistamine Drugs in ARVI Therapy

It is worth noting that the possibilities of pathogenesis-directed therapy for ARVI are characterized by a certain dualism. On the one hand, they are quite broad, due to the similarity of pathogenetic mechanisms in most respiratory viruses. On the other hand, at the same time, they are limited by the differences in pathogenesis that are inherent in each pathogen. Of the most common pathogenetic targets, the most promising is the possibility of influencing IFNs and its receptors, as well as histamine and its H_1_ receptors.

The debate on the necessity of histamine drugs in the treatment of ARVI is still ongoing. For the most part, this ongoing discussion is related to the evidence of AEs of first-generation antihistamine drugs on children’s health. Indeed, their use should be extremely limited. However, due to the development of fundamentally new drugs that are based on polyclonal ATTP Abs to histamine (Ergoferon, Rengalin), we would like to draw attention to some points.

It is well known that the effect of histamine on H₁ receptors underlies a variety of clinical manifestations in allergies; in the upper respiratory tract we find itching, sneezing, swelling of the nasal mucosa and paranasal sinuses, and increased mucus secretion. In bronchial wall swelling, we see hypersecretion and bronchospasm in the lower respiratory tract. In the eye mucosa, we see itching, hyperemia, edema, and lacrimation. H_1_ receptors are not only involved in the early phase of the allergic reaction, but also have immunological properties. As an immunoregulator of inflammation, histamine can enhance the Th1-type response through H₁ receptors [82].

The 2015 Cochrane review presents the results of evaluating the efficacy of antihistamines for monotherapy of the common cold compared with a placebo [83]. In this review, based on 18 RCTs (4342 participants, 212 of whom were children with the common cold—both natural and caused experimentally), convincing data on the efficacy of antihistamines in children were not obtained. However, a Cochrane review of 30 RCTs published in January 2022 (6304 participants; 9 RCTs included children of different ages, 3 RCTs included children from 6 months of age) provided an answer to the question: “Are oral combinations of antihistamines, decongestants, and analgesics effective in the treatment of common cold?” Current evidence suggests some overall benefit from a combination of antihistamines, decongestants, and analgesics in the treatment of the common cold in older children [84].

## 10. Symptomatic ARVI Treatment

Safe and effective symptomatic therapy of ARVI in children is different from that in adults [56]. The symptomatic treatment of ARVI includes management of fever (antipyretics); intoxication and catarrhal nose and nasopharynx symptoms (humectants/elimination drugs, nasal decongestants); as well as throat symptoms and cough.

Among antipyretics in children, paracetamol and ibuprofen have the most favorable safety profile [85]. Ibuprofen has a stronger antipyretic and analgesic effect [56].

Mucous, mucopurulent, or even purulent nasal discharge that lasts for about one week, together with anamnesis and data on the course of disease, indicate an uncomplicated upper respiratory tract ARVI in most children. Antimicrobial therapy is not recommended unless otitis media/sinusitis have been diagnosed [86,87]. If rhinitis is diagnosed, intranasal instillation of physiological saline two to three times a day is effective in removing the mucus, moistening the nasal mucosa, and restoring ciliated epithelium function, as well as relieving cough most associated with an ARVI upper airway cough syndrome/postnasal drip syndrome in children [87,88,89]. The mucolytic acetylcysteine may also be used to reduce a cough in children from 2 years of age—it has been shown to be effective after 6–7 days of administration [56].

The instillation of saline solution up to six times a day reduces nasal secretion and congestion, and lowers the consumption of antipyretics, decongestants, and antibiotics [56]. Severe nasal congestion may be treated with topical nasal decongestants (oxymetazoline, xylometazoline) for short periods of not more than 5 days, with conversion to other means of therapy if symptoms persist.

Among additional means of therapy, camphor, menthol, and eucalyptus ointments have been shown to be effective when spread on the children’s chest/neck before bedtime. These ointments relieve nasal congestion, frequency and severity of nighttime coughs and encourage better sleep [56]. The consumption of honey before bedtime in children over 1 year old may also reduce the frequency and severity of coughing. Herbal remedies such as pelargonium, primrose, thyme, eucalyptus, and ivy leaf extracts may also relieve cough [90].

### Antitussives

The efficacy of antitussives in ARVI in children is debatable. Antitussives can be used to suppress cough; however, there is no strong evidence to support/refute their use [56,91]. The 2017 ACCP (American College of Chest Physicians) guidelines “…suggest against the use of over the counter cough and cold medicines in pediatric patients with cough due to the common cold until they have been shown to make cough less severe or resolve sooner” [92]. However, at the same time, these guidelines and this systematic review noted the antitussive effectiveness of honey was comparable to the effectiveness of the antitussive agent dextromethorphan in children with a common cold. Another review found that neither dextromethorphan nor codeine relieves a cough in children [56]. Codeine-containing drugs for the treatment of cough caused by a cold are not recommended for use in children under 18 years of age [91,92]. Rengalin, the drug based on APTP Abs to bradykinin, histamine, and morphine, has demonstrated therapeutic efficacy in treating cough in children with ARVI. Rengalin reduces the severity and duration of cough, has an anti-inflammatory and anti-edematous effect, and also reduces the irritation of the bronchial mucosa and afferent impulses that trigger a cough reflex, and improves the drainage function of the bronchi, and sputum discharge [93,94].

It is worth noticing that antibiotics are not recommended for ARVI. Uncomplicated ARVI resolve on their own without antibiotic treatment [95]. When treating the common cold, they do not improve the symptoms of ARVI and do not shorten the duration of the disease [96]. The negative impact of the use of antibiotics prevails over their benefits. The AEs of antibiotics are associated with allergic rashes, as well as the development of antibiotic resistance, antibiotic-associated diarrhea, and *C. difficile* infection, which caused severe diarrhea and colitis [96,97].

## 11. Conclusions

Due to the high prevalence, socio-economic burden, and lack of effective control (except for influenza and, partially, RSV infection) of ARVI, they require strong medical attention. The data presented in this descriptive review allows us to conclude that a modern, balanced and evidence-based approach to the choice of ARVI treatments in children should be used in clinical practice. The results of the analysis of current publications given in this article may facilitate the choice of therapy in routine practice, taking into account the capabilities of modern broad-spectrum antiviral drugs that can affect the pathogenesis of ARVI. The published results of clinical trials and systematic reviews with meta-analyses of ARVI in children allow us to conclude that it is possible and expedient to use broad-spectrum antiviral drugs in a complex therapy. This approach can provide an adequate response of the child’s immune system to the virus without limiting the clinical possibilities of using only symptomatic therapy.

## Data Availability

No new data were created or analyzed in this study. Data sharing is not applicable to this article.
